# Genetic Bottlenecks in Time and Space: Reconstructing Invasions from Contemporary and Historical Collections

**DOI:** 10.1371/journal.pone.0106874

**Published:** 2014-09-05

**Authors:** Eleanor E. Dormontt, Michael G. Gardner, Martin F. Breed, James G. Rodger, Peter J. Prentis, Andrew J. Lowe

**Affiliations:** 1 Australian Centre for Evolutionary Biology and Biodiversity, University of Adelaide, Adelaide, South Australia, Australia; 2 School of Biological Sciences, Flinders University, Adelaide, South Australia, Australia; 3 Evolutionary Biology Unit, South Australian Museum, Adelaide, South Australia, Australia; 4 Centre for Invasion Biology, University of KwaZulu-Natal, Pietermaritzburg, South Africa; 5 School of Earth, Environmental and Biological Sciences, Queensland University of Technology, Brisbane, Queensland, Australia; Natural History Museum of Denmark, Denmark

## Abstract

Herbarium accession data offer a useful historical botanical perspective and have been used to track the spread of plant invasions through time and space. Nevertheless, few studies have utilised this resource for genetic analysis to reconstruct a more complete picture of historical invasion dynamics, including the occurrence of separate introduction events. In this study, we combined nuclear and chloroplast microsatellite analyses of contemporary and historical collections of *Senecio madagascariensis*, a globally invasive weed first introduced to Australia *c*. 1918 from its native South Africa. Analysis of nuclear microsatellites, together with temporal spread data and simulations of herbarium voucher sampling, revealed distinct introductions to south-eastern Australia and mid-eastern Australia. Genetic diversity of the south-eastern invasive population was lower than in the native range, but higher than in the mid-eastern invasion. In the invasive range, despite its low resolution, our chloroplast microsatellite data revealed the occurrence of new haplotypes over time, probably as the result of subsequent introduction(s) to Australia from the native range during the latter half of the 20^th^ century. Our work demonstrates how molecular studies of contemporary and historical field collections can be combined to reconstruct a more complete picture of the invasion history of introduced taxa. Further, our study indicates that a survey of contemporary samples only (as undertaken for the majority of invasive species studies) would be insufficient to identify potential source populations and occurrence of multiple introductions.

## Introduction

Biological invasions comprise populations that tend to differ in levels of diversity [1] and invasiveness [2], and invasions by the same species in different areas can be the result of either single or multiple introduction events [3]. Invasive populations can vary in how aggressively they invade and how they respond to control measures [4], and these characteristics can change over time as a result of bottlenecks and multiple introductions [5]. Treating biological invasions as discrete and evolving populations is therefore important both conceptually, for our understanding of the mechanisms behind successful colonisation, and practically, for our capacity to accurately predict and appropriately respond to invasive species.

Biological invasions are generally accompanied by a reduction in genetic diversity in the invasive range [6]. However, multiple introductions are very common in human-mediated introductions [7]–[11], and can augment genetic diversity [12], increase propagule pressure and reduce mate limitation [10]. High genetic diversity can be beneficial on both ecological and evolutionary timescales: in the short term, high diversity has been shown to improve colonisation success [13]. In the longer term, admixture between disparate source populations can reduce inbreeding depression [11] and increase fitness [14], as well as produce novel gene combinations and increase evolutionary potential [15]–[17]. Conversely, multiple introductions can sometimes result in a ‘mosaic of maladaptation’ [6], where populations would be better adapted to different locales, but are limited by the spatially stochastic nature of their introduction and restricted gene flow.

What processes are acting during the lag phase of an introduction, where species are present in a new environment but not yet invasive, is a critical question in invasion biology [18]. A major problem in studying lag phase processes is that once a species has become invasive, the opportunity to study lag phase dynamics in real time has passed. However, herbarium material is an important resource that potentially documents a species' history throughout the lag phase period. Herbarium records are often used to plot the spread of invasive species through time (e.g. [19]–[21]), and investigate enemy release [22], changes in morphological traits over time [23] and genetic diversity and introduction sources [24]. However, by combining the temporal information from herbarium records with modern advances in DNA extraction and genetic analysis, we can study the genetic composition of invasions through time. These techniques have been used successfully to detect cryptic invasions [25], investigate historical genetic structure in the native range prior to introduction [26], document the accumulation of genetic diversity over the course of an invasion [27] and identify adaptation rapid adaptation [28]. This approach could also help clarify the role of multiple introductions in successful invasions, and how these processes relate to lag phase.

*Senecio madagascariensis* Poir. (Asteraceae) is a diploid herbaceous perennial plant native to Southern Africa and invasive in several countries, including Australia. The first herbarium record for the species in Australia was lodged in 1918 and collections exist to the present day. The species is classified as a noxious weed in the state of New South Wales and is estimated to cost farmers *ca.* AU$2.7 million per year [29]. Although recognised as a weed in New South Wales in the 1960s [30], *S. madagascariensis* was present in Australia for approximately 70 years before rapid population expansion in the 1980s [31], constituting a considerable lag phase. More recent work has identified a reduction in molecular transducer gene expression (often associated with response to biotic stimuli) in contemporary Australian *S. madagascariensis* compared to material from South Africa [32]. This finding suggests dramatic genetic changes may have occurred in invasive populations during lag phase, subsequently aiding the rapid spread observed during the 1980s [32]. Specifically, a reduction in expression of genes involved in response to biotic stimuli could be indicative of enemy release in the invasive range and potentially the evolution of increased competitive ability [33]; although research into the herbivore community composition of *S. madagascariensis* in Australia has revealed a complex relationship over time [34]. An alternative explanation might be that a more invasive strain of *S. madagascariensis* was subsequently introduced around the time of lag phase break, and was then able to spread more effectively than the resident *S. madagascariensis* genotypes present at that time. This second scenario has been supported in a study of the European invasion of *S. inaequidens*, where historical and molecular data were combined to reveal that a 70 year lag phase in Bremen, Germany, was broken by the arrival of additional native range genotypes via a different invasion route, which ‘overran’ the more slowly expanding resident population [35]. Recent work examining the dispersal ability of *S. madagascariensis* populations at the centre verses edges of its range in Australia have not found any significant differences [36], however this does not preclude superior dispersal ability across the range in Australia when compared to native or historically invasive populations. Gaining a greater understanding of the spatial, temporal and genotypic dynamics of *S. madagascariensis* over the course of the Australian invasion will increase our understanding of the circumstances surrounding its break from lag phase.

**Figure 1:**
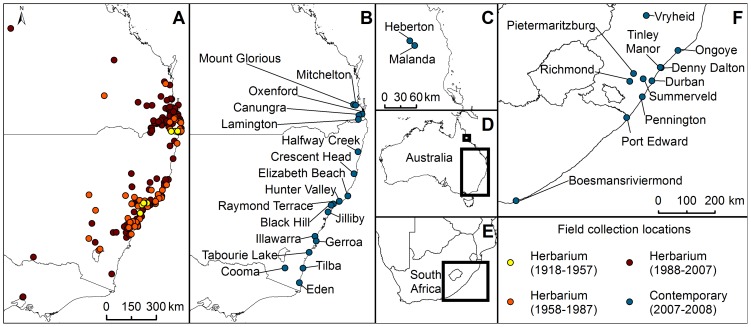
Senecio madagascariensis sampling locations. Geographic locations of: herbarium records sampled in Australia (A); contemporary collections in Australia (B, C) and South Africa (F). Extent maps indicating sampling areas in country-wide context (D, E).

Our study combines traditional herbarium record mapping with genetic analyses of both historical and contemporary collections of *S. madagascariensis* in Australia, and an analysis of genetic variation in contemporary samples from its native range in South Africa (Fig. 1). Specifically we aimed to explore whether: (a) the Australian invasion is comprised of a single panmictic or multiple independent populations; (b) genetic diversity in the native range differed significantly from that of the Australian population(s); (c) multiple introductions and/or multiple source populations can be located.

## Materials and Methods

### Study species

*Senecio madagascariensis* Poir. (Asteraceae) is an herbaceous plant growing to around 0.6 m with green leaves and bright yellow inflorescences. Flowering occurs predominantly in spring and autumn and flowers are insect pollinated. The species is a diploid (2*n* = 20), obligate outcrosser and its seeds are wind dispersed. *Senecio madagascariensis* is native to South Africa (where it is widespread throughout the coastal provinces of the Western Cape, Eastern Cape and KwaZulu-Natal) and Madagascar [37], [38]. It also has limited native populations in Swaziland and Mozambique (Invasive species compendium, www.cabi.org/isc).

*Senecio madagascariensis* is thought to have been introduced to Australia from the dry ballast of ships trading between Europe and Australia via South Africa [30]. Originally prominent in the New South Wales (NSW) Hunter Valley (the first herbarium specimen was found in 1918 at S 32° 43′, E 151° 45′), anecdotal evidence points to the transportation of *S. madagascariensis* to north coast NSW in crop seed *c.* 1940 [39] (the first north coast NSW herbarium specimen was found in 1948 at S 28° 49′, E 153° 16′). Currently, *S. madagascariensis* is present all along the coast of NSW and into south-east Queensland. Plants at two sites in Far North Queensland (FNQ) have also been recently identified as *S. madagascariensis*, confirming the predictions identifying FNQ as climatically suitable for the species [40].

### Contemporary field collections

The most likely native provenance of *S. madagascariensis* in Australia has been narrowed down to South Africa by ITS1 sequence data comparisons between Australian samples and those from South Africa (KwaZulu-Natal province, KZN) and Madagascar [41]. Provenance has been further identified to KZN by morphological and isozyme data which included individuals sampled from the Western Cape, Eastern Cape and KZN provinces in South Africa, Swaziland and Madagascar [38]. We therefore concentrated our sampling on KZN (11 sites). We also sampled from the Eastern Cape and Western Cape provinces (four sites) as these were highlighted as more distantly related to Australian fireweed [38]. A representative voucher specimen was lodged from each South African site to confirm species identity (this can be particularly challenging in South Africa where many similar *Senecio* species co-exist). Only one population of the four collected outside of KZN was included in the final analysis due to misidentification in the field and polyploidy (see results).

In Australia, 20 sites were sampled across the known distribution of *S. madagascariensis*. As misidentification is less likely in Australia (the native *S. pinnatifolius* is superficially similar but easy to distinguish based on bract number), a single voucher specimen from Halfway Creek was lodged to confirm identity. All sites included in our study had their voucher specimen confirmed as *S. madagascariensis* by a taxonomist (see acknowledgements). Voucher details are in Table S1. Fresh leaf samples from *ca.* 20 plants ≥5 m apart at each site were collected (Table S2). Leaves were immediately placed on silica gel and stored separately until DNA extraction.

### Herbarium collections

Electronic records were obtained from all Australian herbaria for *S. madagascariensis* and collated into a single database. The coordinate points for each observation were checked against Google Earth v4.1 (Google Inc.) and all duplicate records were removed. The density of herbarium collections was visualised using the density tool in ArcMap v10.0 (ESRI). Physical sampling of herbarium vouchers was undertaken for all *S. madagascariensis* accessions kept at the Queensland Herbarium, National Herbarium of Victoria and National Herbarium of New South Wales. Duplicate records containing different plants collected at the same time from the same location were included in order to capture as much potential diversity as possible (*n* = 247 sampled and DNA extracted, *n* = 223 successfully genotyped at all loci). A small leaf sample was taken from each record and stored at room temperature until DNA extraction.

### Microsatellite genotyping

DNA extraction was carried out using the Machery-Nagel Nucleospin Plant II Kit with the PL2/PL3 buffer system. Primers for nine previously published nuclear microsatellite loci [42] were used to screen all native and invasive contemporary collections of *S. madagascariensis*. Previous trials using nuclear microsatellites with DNA extracted from herbarium voucher specimens achieved <10% successful amplification (unpublished data), possibly due to low copy number of nuclear DNA compared to chloroplast DNA which produced >90% successful amplifications. Nuclear microsatellite analyses were therefore restricted to contemporary collections only. PCR reactions (10 µL) were prepared with *ca.* 20 ng of template DNA, 1x reaction buffer, 0.2 mM of each dNTP, 2.5 mM MgCl_2_, 0.4 µM of each primer, and 0.02 U Amplitaq Gold (Applied Biosystems). PCR reactions were carried out with an initial denaturation step of 94°C for 2 min, 35 cycles of 94°C for 1 min, *T_a_* °C for 1 mins, 72°C for 1 min 30 s, and a final extension at 72°C for 30 min. See Table S3 for annealing temperatures (*T_a_*). Products were separated using the ABI 3730 DNA analyzer (Applied Biosystems) with the GeneScan – 500 LIZ size standard. Alleles were automatically called using GeneMapper software (Applied Biosystems) and double-checked manually.

Nine of the ten previously published chloroplast microsatellite primer pairs for *Senecio madagascariensis* [43] were assessed for polymorphism using one individual from each of the native sites sampled (the Se-76 locus was excluded as its fragment sizes were >500 bp and therefore unreliably measured with our size standard). 10 µL PCR reactions were prepared with *ca.* 20 ng of template DNA, 1x reaction buffer, 0.2 mM of each dNTP, 2.5 mM MgCl_2_, 0.5 µM of each primer, and 1 U IMMOLASE DNA polymerase (Bioline). Reactions were carried out with an initial denaturation step of 94°C for 5 min, 30 cycles of 94°C for 20 s, 50°C for 20 s, 72°C for 20 s, and a final extension at 72°C for 30 min. Products were analysed and scored as above. All contemporary South African samples and all Australian herbarium accessions were screened (Table S4) with three identified polymorphic loci. Details of all loci included in the final analyses are listed in Table S3. PCR reactions were repeated for *ca.* 10% of samples in order to calculate error rates.

### Data analysis

All microsatellite loci were assessed for suitability (Appendix S1). Genetic clusters in Hardy Weinberg (HW) equilibrium were determined using the program structure v2.3.3 [44] using an admixture model. structure works by assigning individuals to populations based on clusters of individuals with gene frequencies in HW and linkage equilibrium, using a Bayesian, model-based algorithm. Each run consisted of a burn-in period of 100,000 Markov Chain Monte Carlo (MCMC) repetitions, followed by 1,000,000 MCMC repetitions. Possible numbers of discrete populations (*K*) were set from one to the maximum number of sites sampled. Each value of *K* had five separate runs to allow detection of any spurious results. structure was run for all sites combined (South Africa and Australia) and separately for the native (South African) and invasive (Australian) ranges. The most likely value of *K* was determined using structure harvester 0.6.93 [45] and taken as the value of *K* at which Δ*K* is maximal [46]. Structure outputs were averaged over runs using clumpp [47] and displayed with distruct [48].

Appropriate measures of population differentiation have been a contentious issue in the literature (e.g.[49]–[53]). Meirmans and Hedrick [52] suggest reporting *F_ST_* along with *F'_ST_* or *D_est_*, and we have chosen to report all three statistics to maximise future comparability of our results with other studies. *F_ST_* and *F'_ST_* were calculated using fstat v2.9.3.2 [54], the later in combination with recodedata v0.1 [55] which creates a dataset that maximises possible *F_ST_* values, enabling the *F_ST_* value obtained for the original data to be scaled to its theoretical maximum. The program smogd v1.2.5 [56] was used to determine *D_est_*, the particular algorithm used is unable to use groups of individuals with missing data for an entire locus, so three Australian populations were excluded from the calculation (Table S2). Isolation-by-distance was measured using a Mantel test with 9,999 permutations between pairwise *F_ST_* values and geographic distance (the Euclidean distance between population latitudes and longitudes) in genalex v6.4 [57], [58].

Observed heterozygosity (*H_o_*), unbiased expected heterozygosity (*H_e_*), mean number of alleles per locus (*A*) and the inbreeding coefficient (*F_IS_*) were calculated using genalex v6.4 [57], [58]. Allelic richness (*A_r_*) and private allelic richness (*A_pr_*) [59] were calculated using adze v1.0 [60]. The inbreeding coefficient, corrected for null alleles (*F_IS-c_*), was calculated with inest2.0 [61]. Differences between populations, as determined by genetic clusters in structure, were investigated using the Excel Template for Kruskal-Wallis test (with Dunn's posthoc Test) (Gianmarco Alberti). Range-wide comparisons (native vs. invasive) were analysed with Mann-Whitney U tests [62].

For the chloroplast microsatellite dataset, each unique combination of alleles was defined as a separate haplotype. Counts were made of total number of haplotypes and private haplotypes in a particular area. Simpson's diversity index (*H*) was determined in contrib v1.02 [63] using the following equation:

*H* = *n*/*n*–1(1−∑*_i_ x_i_^2^*) where *x_i_* is the estimated frequency of the *i*th haplotype in the population when a sample of *n* individuals is drawn at random [64]. Haplotypic richness (*R_h_*) was calculated in contrib v1.02 [63] and rarefied to sample size *n* = 11. Haplotype data were divided into three groups based on collection date (1918–1957, 1958–1987 and 1988–2007) to examine changes over time.

To assess potential source populations in South Africa, the proportion of haplotypes found in Australia in 1957 were compared with those found in contemporary native sites using an extension of the Fisher exact test [65]. A median-joining network [66] was constructed using length differences for the chloroplast microsatellite dataset in network v4.6.0.0 [67].

### Simulations

To assess whether the patterns of haplotype emergence and diversity in the Australian herbarium record could be explained by random sampling, we simulated various introduction hypotheses (Table S5) in Resampling Stats Add-In for Excel v4.0 (statistics.com), using 10,000 repeat samples to obtain estimated *P* values in each case. For simulation details see results. For one simulation we used the ‘Calculate Geodesic Distance Between Points’ model for ArcGIS v10.0 (ESRI) available from the ‘Geoprocessing Model and Script Tool Gallery’ (resources.arcgis.com) to calculate the distance between each herbarium specimen location and all major ports in the study area (major ports were chosen based on the assignment of ‘medium’, ‘large’ or ‘very large’ on worldportsource.com) (Table S6).

### Ethics statement

All relevant permits and approvals were obtained for the work presented in this study. Herbarium records were accessed and sampled with permission from the Council of Heads of Australian Herbaria, custodian of Australia's Virtual Herbarium. Population samples were taken from public land or with consent from the landowner. No protected species were sampled.

### Data access

A genalex formatted excel file [57], [58] containing all microsatellite allele calls for all individuals in all populations can be found at Appendix S2.

## Results

**Figure 2:**
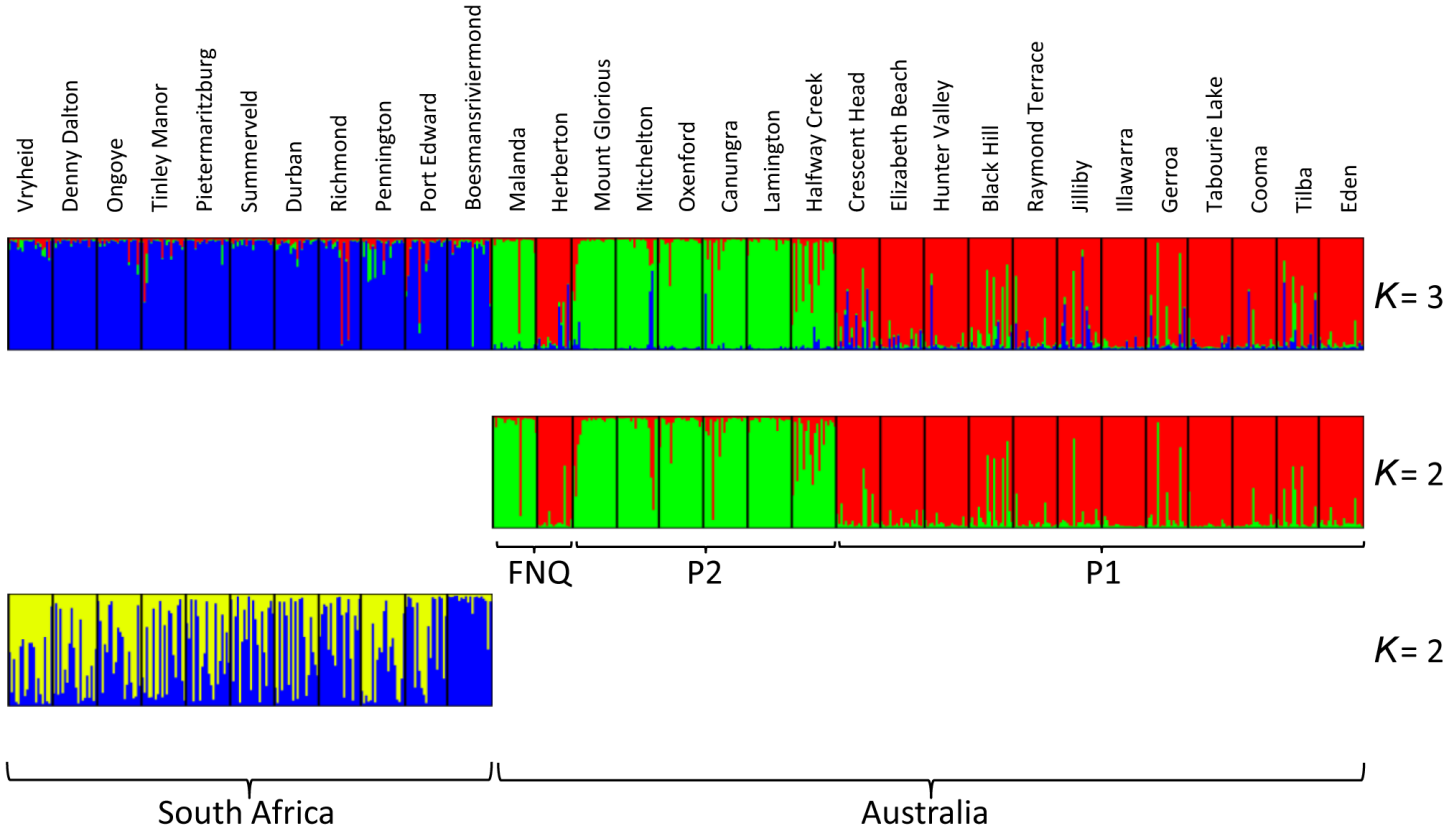
Results of structure analyses. Graphical outputs of all structure analyses undertaken; all samples (top) showing K = 3 genetic clusters; Australia only (middle) showing K = 2 clusters; and South Africa only (bottom) showing K = 2 clusters. Sampling site names are listed above their respective outputs.

**Figure 3:**
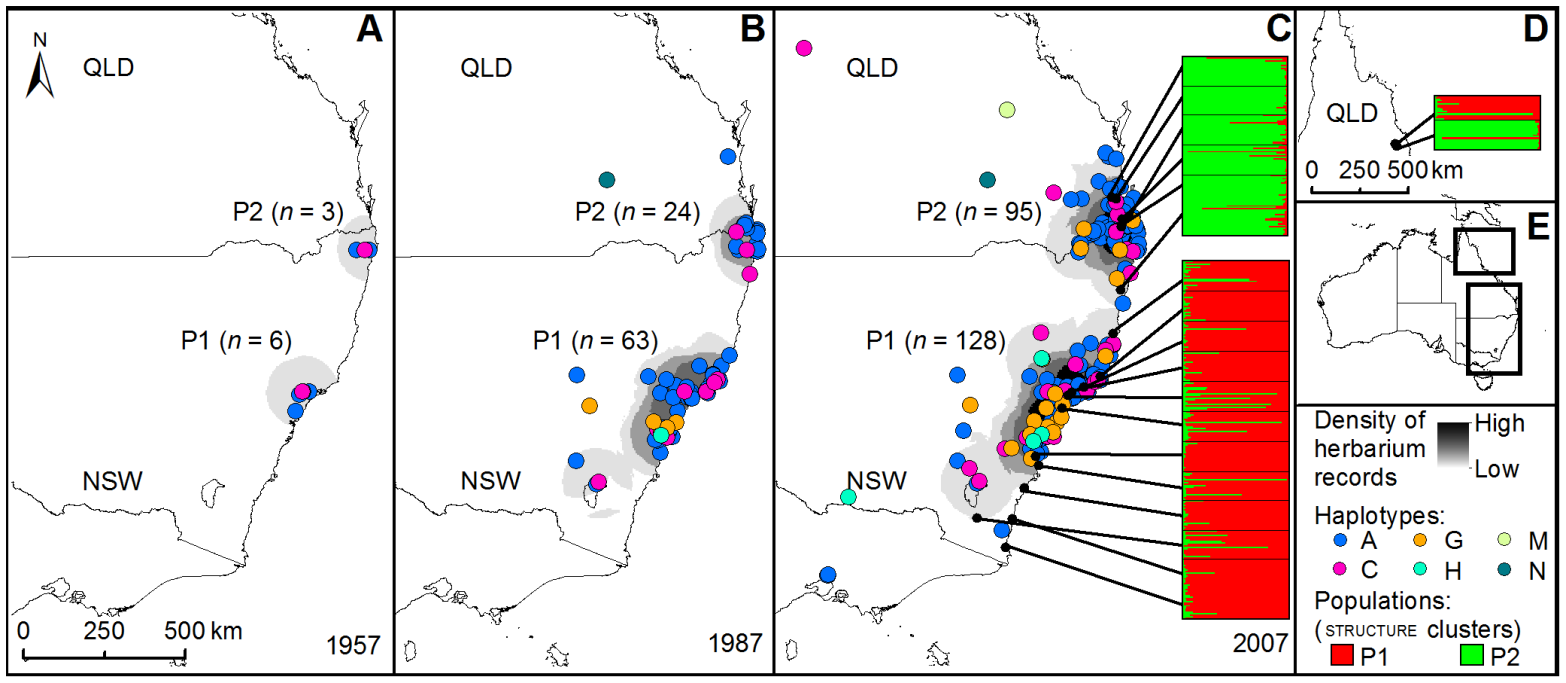
Maps illustrating the spread of Senecio madagascariensis in Australia through time. Density of herbarium records and location of chloroplast haplotypes (A-C); location of P1 and P2 derived from nuclear microsatellite data from contemporary field collections (as defined by clusters in the program structure [44]) (C, D); clustering of sites in Far North Queensland with P1 and P2 (D); extent of maps in relation to Australia as a whole (E).

All nine nuclear microsatellite loci were evaluated and seven deemed appropriate for use in further analyses. Two were excluded due to inconsistent banding patterns (Appendix S1) and these same two loci were also excluded from Le Roux et al. [68] analysis of *Senecio madagascariensis* in Hawaii. Samples from one South African site (Hluhluwe, KZN; Table S1) were obviously polyploid based on *n*>2 alleles present for several loci. As *S. madagascariensis* in Australia is exclusively diploid [69], this population was excluded from further analyses. structure results for all individuals are presented in Figure 2; two distinct Australian populations (i.e. genetic clusters) were determined on the basis of nuclear microsatellites when data from Australia only were considered (*K* = 2). When South African data were included in the analysis, the same Australian clusters were defined and all South African material clustered together independently (*K* = 3). When considered independently, South Africa was partitioned into two populations (*K* = 2), with Boesmansriviermond from the Eastern Cape representing a distinct cluster and all other sites from KwaZulu Natal comprised of individuals assigning to both clusters. The Australian clusters roughly equated to a south-eastern population (P1), ranging between Eden and Crescent Head in New South Wales (NSW), and a mid-eastern population (P2), ranging from Halfway Creek in NSW to Mount Glorious in Queensland (QLD) (Fig. 3).

Mapping the spread of invasion from herbarium records in Australia showed an original invasion focus at Raymond Terrace, lower north coast NSW in 1918, with a second invasion focus *ca.* 1948 at Lismore in northern NSW. The invasion continued to spread from these two foci up to the present day (Fig. 3). Fifty eight percent of all alleles were found in both South Africa and Australia, 33% were unique to South Africa and 9% unique to Australia. Within Australia, 38% and 4% of alleles were unique to P1 and P2 respectively. Ten alleles (4% of the global total) were found in P1 and nowhere else. One allele (0.4% of the global total) was found exclusively in P2.

Of the two sites from Far North Queensland (FNQ) included in the study, Malanda clustered with P1 whilst Herberton clustered with P2, despite these sites being only *ca.* 20 km apart (Fig. 3). These two sites are *ca.* 1300 km from the next closest plants in QLD and so are effectively isolated from the main invasion. These very large geographic distances mean that these two sites can have no gene flow with their parental populations, and therefore they constitute new invasion foci in Australia. Consequently, they have been excluded from population level comparisons of P1 and P2 and analyses of isolation-by-distance.

Significant isolation-by-distance was detected across the two Australian populations (*r* = 0.63, *P*<0.01) with distinct clustering of within and between population comparisons (Figure S1), but was not evident when P1 (*r* = −0.03, *P* = 0.507) and P2 (*r* = −0.17, *P* = 0.418) were considered independently. Isolation-by-distance was evident in South Africa with inclusion of Boesmansriviermond (*r* = 0.81, *P* = 0.039), but was not significant when Boesmansriviermond was excluded (*r* = 0.07, *P* = 0.364).

### Population level comparisons

There were significant differences in all measures of diversity based on the nuclear microsatellites data between South Africa, P1 and P2 (Table S7). In all cases, diversity was highest in South Africa, followed by P1 and then P2 (Table S2). Post-hoc tests confirmed that either South Africa resulted in significantly higher values than P1 and P2 (for measures of *A*, *A_pr_* and *H_e_*), or that P2 resulted in significantly lower values than P1 and South Africa (for measures of *A_r_* and *H_o_*) (Table S7). Comparing the native and invasive areas as a whole, South Africa had significantly higher levels of genetic diversity than Australia in all metrics (Mann-Whitney U test: *A*, *U* = 211.5, *P*<0.001; *A_r_*, *U* = 191.0, *P*<0.001; *A_pr_*, *U* = 201.0, *P*<0.001; *H_o_*, *U* = 167.5, *P*<0.05; *H_e_*, *U* = 203.5, *P*<0.001).

Population differentiation was lowest in South Africa (*F_ST_* = 0.044), slightly higher in P1 (*F_ST_* = 0.049) and considerably higher again in P2 (*F_ST_* = 0.081). The largest value of *F_ST_* was obtained from Australia as a whole, and globally (both *F_ST_* = 0.100). Other differentiation statistics are reported for comparison (Table S2). No significant difference in inbreeding (as measured by *F_IS_* and *F_IS-c_*) were detected between populations (*F_IS_*: *H_c_* = 5.156, *P* = 0.076; *F_IS-c_*: *H_c_* = 2.592, *P* = 0.274) or ranges (*F_IS_*: *U* = 118, *P* = 0.760; *F_IS-c_*: *U* = 69, *P* = 0.095).

**Figure 4:**
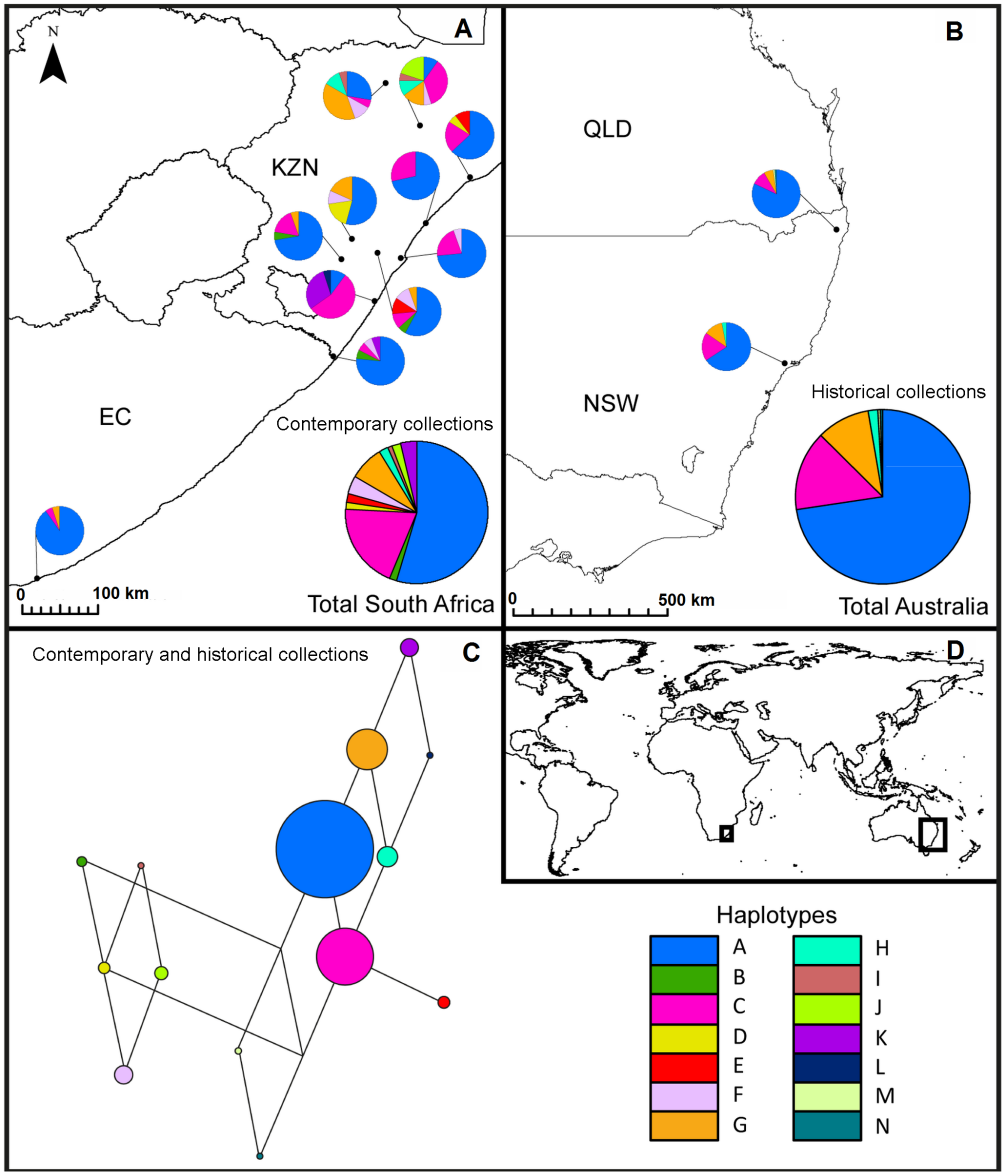
Location of Senecio madagascariensis haplotypes based on three chloroplast microsatellite loci in contemporary samples from South Africa and historical samples from Australia. The proportion of haplotypes found at each sampled site in South Africa (A); haplotypes from all herbarium records in Australia, according to their position in either the south-eastern Australian population P1 or mid-eastern Australian population P2 (B). Size of pie charts are proportional to the number of individuals sampled; median joining network of S. madagascariensis, where the smallest connector length represents one character change (C); map extents (D). Colour codes for haplotypes are consistent throughout.

Complete cpSSR haplotypes (successful amplification at all three loci) were obtained for 223 herbarium samples from Australia and 195 contemporary samples from South Africa (Table S4). Individual cpSSR alleles failed to amplify in 4.5% of reactions. Failure rate was always greater in the herbarium samples compared to contemporary samples (cpSSR1, 0.00 vs 0.04; cpSSR2, 0.09 vs 0.12; cpSSR5, <0.01 vs 0.02). Individual herbarium specimens were grouped into two populations based on whether they were found within the geographic range of P1 or P2. Of the global total sampled in this study, eight chloroplast haplotypes (57%) were unique to South Africa. Two haplotypes (14%) were unique to Australia. These two unique haplotypes occurred only once each in P2 (near Warkon, QLD and Theodore, QLD) and not in P1 at all. P1 included one haplotype not present in P2. Australia and South Africa were both dominated by two closely related haplotypes, A and C; a rarer haplotype H, found in P1 in Australia, was only found in adjacent inland sites in South Africa (Table S4, Fig. 4). Haplotype data for the native range was analysed at the site level, and for South Africa as a whole (Fig. 4). Both *H* and *R_h_* were greatest in South Africa, followed by P1 then P2 (Table S4). Haplotype frequencies found in Australia in 1957 were most similar to those in contemporary populations at Tinley Manor and Durban. Haplotype frequencies were significantly different between Australia in 1957 and Vryheid (*P*<0.01), Denny Dalton (*P*<0.001) and Pennington (*P*<0.001). As no contemporary collections in Australia or historical collections from South Africa were screened for cpSSRs, comparisons of these cpSSR data should be interpreted with caution.

### Simulations

We simulated various introduction scenarios to assess whether the patterns of haplotype emergence and diversity in the Australian herbarium record could be explained by random sampling. The null hypotheses tested are summarised in Table S5. We simulated random sampling of nine individuals (without replacement) from South Africa (representing the number of herbarium specimens collected in Australia by 1957) and tested the probability of obtaining larger differences in haplotypic proportions between South Africa and this sample than were observed in our dataset. The difference was not significant (*P* = 0.633). A similar simulation based on 83 individuals (representing the number of herbarium specimens collected in Australia by 1987) was significant (*P*<0.01), which indicates that the haplotype composition in Australia at that time no longer constituted a random sample of those found in the native area studied.

New haplotypes appear in the herbarium record in 1975 (haplotype G) and 1983 (haplotype H). To test whether the absence of these haplotypes in the herbarium record prior to 1975 was potentially a sampling effect, we simulated random sampling (with replacement) of 32 individuals (number of herbarium records collected prior to the appearance of haplotype G in 1975) from a pool of 223 (total number of herbarium records genotyped in Australia) and asked how often the sample did not contain either haplotype G or H. The simulation indicated that the absence of these haplotypes in the early herbarium records is unlikely to be a sampling effect (*P*<0.05).

Haplotypes G and H appeared in the herbarium record for the first time <35 km from the port of Sydney. To test whether this proximity of new haplotypes to the same major port could be attributed to random sampling, we simulated sampling one from a pool of the first 31 different locations recorded in Australia (the number of different herbarium record locations when the first individual with haplotype G was recorded). We also sampled a second individual from a pool of the first 49 different locations recorded in Australia (the number of different herbarium record locations when the first individual with haplotype H was recorded). We compared the distances from major ports of these two samples and recorded if they both fell within 35 km of the same port. It was unlikely that the proximity of these samples to a major port in Australia was the result of random sampling from the herbarium record (*P*<0.05).

To assess whether our genetic data supported the anecdotal evidence that P2 was founded from material originating from P1, we asked whether random independent sampling from the native range could produce two populations in which the second, smaller one contained fewer private haplotypes than those found in the non-simulated data for P2. In other words, apart from the unique haplotypes M and N (which may be erroneous) P2 appears to be comprised of a subset of those haplotypes present in P1, a situation consistent with the founding of P2 from P1. We sought to test whether this scenario was likely to arise by chance, through random sampling of native haplotypes. To achieve this we simulated random sampling (with replacement) of 223 individuals (total number of herbarium records genotyped in Australia) and assigned them to two simulated populations (sP1 and sP2). We assessed the proportion of haplotype occurrences that appeared in sP2, but not sP1, and compared this to the actual proportion observed in the data (0.021, caused by the occurrence of haplotypes M and N in P2). The observed data could be explained by independent native range introductions (*P* = 0.813), and so is inconclusive in distinguishing between these two potential scenarios. Our simulation approach did not incorporate random sampling over multiple generations and hence does not account for the potential effects of genetic drift.

## Discussion

By combining a detailed microsatellite study of contemporary and historical collections with spread data from herbarium records, we find consistent evidence that the introduction of invasive *Senecio madagascariensis* into Australia was founded in lower north coast New South Wales (NSW, P1). A second, genetically depauperate invasion focus was also founded in northern NSW (P2). Anecdotal evidence suggests that this second introduction occurred later from within Australia, and our results are consistent with that interpretation. However, a separate introduction event from the native range cannot be ruled out, and the timing of such an event cannot be determined. Despite the relatively low resolution of the cpSSR data set, genetic analysis of herbarium specimens from Australia indicates that the number of unique haplotypes has increased over time, probably as a result of subsequent introduction(s) due to the close proximity of these new haplotypes to the port of Sydney. The timing of additional introduction(s) (some time prior to 1983) was likely to be shortly before the reported end of lag phase of *S. madagascariensis* in 1988 [31]. Therefore, it remains plausible that *S. madagascariensis* emerged from lag phase to become invasive due to the introduction of additional material from the native range. Subsequent introductions could have triggered invasiveness, either by means of increased standing genetic diversity on which selection could act, or by the introduction of better adapted genotypes more readily able to spread in the Australian environment. Nevertheless, our results do not rule out other explanations for the success of *S. madagascariensis* in Australia, such as enemy release ([32], but see [70]).

Population structure analysis found evidence for two distinct populations in Australia, (P1 and P2). This division is supported by significant isolation-by-distance across Australia as a whole, but not within P1 or P2, greater differentiation across Australia than in South Africa, and by the spread of herbarium records. P2 appears to comprise a subset of the genetic diversity found in P1, with very low occurrence of private alleles and private haplotypes, supporting the view that P1 was the primary source of the secondary invasion at P2. This scenario is also consistent with anecdotal evidence that *S. madagascariensis* was transported in crop seed to north coast NSW in the 1940s [39]. However, our simulations indicated that a null hypothesis of (an) independent introduction(s) from the native range founding P2, cannot be rejected and so a definitive conclusion cannot be drawn from the present data.

Reduction in the genetic diversity of *S. madagascariensis* upon introduction to Australia does not appear to have hindered its spread. Levels of diversity observed for *S. madagascariensis* in Hawaii [68] were less than those observed in Australia and the native range in South Africa (e.g. *H_o_*: Hawaii  = 0.43; Australia  = 0.55; South Africa  = 0.61). These results support the pattern of reduced diversity in biological invasions [6]. A species' capacity to thrive across a broad range of environmental conditions, despite limited genetic diversity, could be due to high levels of phenotypic plasticity [8], changes in gene expression [32] or that diversity may simply be ‘high enough’ for populations to adapt to the new conditions. Alternatively, the invasive range may impose only weak selection pressures allowing relatively genetically depauperate populations to thrive.

Here we report genetic differentiation via *F_ST_*, *F'_ST_* and *D_est_*. Values for *F_ST_* were low (maximum  = 0.1), generally indicating weak population differentiation. However, *F_ST_* is sensitive to within population variation and where this differs between regions (i.e. native and invaded ranges) problems develop in accurately interpreting *F_ST_* results. The use of *F'_ST_* should circumvent this problem by scaling *F_ST_* to its maximum possible value [52]. Jost's *D*, on the other hand [50], is based on the effective number of alleles and not heterozygosity, and is not sensitive to within population variation. In our study, these statistics are generally consistent, however they vary widely for their estimation of differentiation in P2, reflecting the different processes best described by these statistics. *F_ST_* is a ratio of genetic variances [71] and a higher value indicates a reduction in expected heterozygosity relative to the total population. The differences observed between *F_ST_* and *F'_ST_* are explained by the scaling factor, making *F'_ST_* more appropriate for comparing differentiation between regions. In contrast, *D_est_* indicates the level of allelic differentiation between populations and the low result suggests that sites within P2 have very similar allelic composition. As expected heterozygosity is independent of the exact allelic composition of populations, these differences can be simultaneously observed in the same population (here P2).

We found evidence for significant inbreeding (as measured by *F_IS_*) in all populations. This result is surprising since *S. madagascariensis* is considered an obligate outcrosser [72], [73]. The potential for high null allele rates in the dataset is likely to have artificially decreased observed heterozygosity, resulting in higher values of *F_IS_*. The inbreeding coefficient corrected for null alleles (*F_IS-c_*) indicates levels of inbreeding an order of magnitude lower than the uncorrected *F_IS_*, as would be predicted by the outcrossing mating system. In the original primer note [42], half of the loci used in this study exhibited significant heterozygote deficiencies, and in a study of *Senecio madagascariensis* in Hawaii, heterozygote deficiencies were also detected in most study populations [68], although null alleles were not explicitly considered as a contributory factor. Breeding between close relatives (not strictly selfing) would also contribute to higher levels of *F_IS_* within populations and could possibly be the result of higher levels of relatedness between individuals in the founding populations. Maintenance of self-incompatibility, but increased mate availability, has been identified in the closely related *S. inaequidens* [74]. Lafuma and Maurice [74], postulated that an increase in the average level of dominance relationship between S-alleles that control self-incompatibility could have allowed *S. inaequidens* to retain selfing avoidance while reducing the disadvantage of limited mate availability. A similar scenario could have occurred in *S. madagascariensis* in Australia, however if this was the case, we might expect significant differences between native and invasive populations in measures of inbreeding, which was not evident in the current data set.

The appearance of new haplotypes in the herbarium record could be the result of homoplasic mutations (regeneration of identical native range haplotypes) within Australia, or very low initial haplotype frequencies leading to evasion of herbarium sampling. Our simulations indicated that the chance of low frequency haplotypes (G and H) being missed in herbarium sampling is sufficiently small as to be unlikely (*P*<0.05), making a secondary introduction of material including these new haplotypes a more likely explanation. However, our simulations presume that haplotypic proportions have remained steady over the course of the invasion, an assumption that may not hold true, particularly if significant genetic drift has occurred or if selection has acted to increase the frequency of particular haplotypes over time. The proximity of first occurrences of haplotypes G and H to the port of Sydney suggests the arrival of additional material containing these haplotypes through Sydney. In support of this scenario, our simulations indicate that the chance of two new haplotypes occurring in the herbarium record within this range of a major port merely by chance is sufficiently low to also make it an unlikely explanation (*P*<0.05), supporting additional introduction(s) as a more parsimonious explanation.

Locations of the haplotypes present in South Africa indicate that the two oldest and most common haplotypes in Australia (A and C) were also found in the majority of native sites, making identification of the initial source of introduction challenging. Both Tinley Manor and Durban have a very similar haplotypic composition to Australia in 1957, and Durban is the largest port in South Africa in terms of shipping volume, providing a potential invasion pathway. Of the rarer haplotypes in Australia (G and H), which appear to have been introduced later through Sydney, H only occurs at two of the native sites sampled (Vryheid and Denny Dalton), which are within 80 km of each other in the Zululand District Municipality. The restriction of this haplotype to a specific native area suggests that the area may have been a source for *S. madagascariensis* invasion in Australia. However, it is also possible that other native areas harbour this haplotype and these areas were not sampled in our study. The similarity between Tinley Manor, Durban and Australia in 1957, with regards to haplotypic composition, as well as the significant difference between the haplotypic composition of Australia in 1957 and the Zululand sites, suggests that an initial introduction from the Durban area (of common haplotypes A and C) was later followed by introduction from Zululand, including haplotypes G and H. However, these results must be treated with caution as the haplotype frequencies in 1957 in Australia may not necessarily mirror those of the source area today, due to genetic drift, selection in South Africa and/or selection in Australia since *S. madagascariensis* was introduced.

Previous efforts to manage *S. madagascariensis* in Australia have led to an unsuccessful search for biological control agents in Madagascar [75]. Failure of the biocontrol program was likely due to poor adaptation of Madagascan agents to *S. madagascariensis* genotypes in Australia, as these genotypes most likely originated from South Africa ([38], [41]; results presented here). Our work builds on these previous findings to suggest that the areas around Durban and Zululand in KwaZulu-Natal may be good areas for biological control prospecting.

Using our nuclear microsatellite dataset, we were unable to conclusively test for admixture between materials originating from disparate native sources. Clustering analysis found greatest support for two South African populations, with differentiation of the Eastern Cape but mixed population origins of all sites in KwaZulu Natal. This differentiation of the Eastern Cape site is corroborated by significant isolation-by-distance in the native range only when the Eastern Cape site was included. This genetic homogeneity in the native range made source identification impossible from the nuclear microsatellite dataset, a common problem when native range *F_ST_* is low [76]. The two Australian populations clustered separately from the single South African population when all samples were analysed together, possibly due to drift post-introduction [77], or because our sampling was not wide enough to include the true source population(s). Further sampling in the native range, including herbarium accessions [26], may therefore lead to identification of more likely sources.

## Conclusions

Our study successfully combined genetic analysis of contemporary field and historical herbarium collections to reconstruct the history of *Senecio madagascariensis* in Australia, from introduction, through lag phase and into the recent period of invasion. By combining these different resources we emphasise how a survey of contemporary samples only (as undertaken for the majority of invasive species studies) would have failed to identify possible source populations and multiple introductions. Using this approach to reconstruct a more complete picture of the invasion history of introduced taxa will improve our understanding of invasion pathways and lag phase processes, shed further light on the role of multiple introductions, and potentially pave the way for more effective control of invasive species.

## Supporting Information

Figure S1 **Isolation by distance between and within populations.** Linearised F*_ST_* values (F*_ST_*/(1-F*_ST_*)) regressed on lat-long Euclidean distance between pairs of sampled sites. Shading shows the effects of between and within population comparisons. Significant isolation-by-distance (Mantel's test P<0.01; r = 0.63) was detected across Australia as a whole.
(PDF)

Table S1 ***Senecio madagascariensis*** **herbarium voucher details.**
(PDF)

Table S2 **Location details and diversity metrics of all sites sampled for nuclear microsatellite analysis.** Population locations: KZN, KwaZulu-Natal; EC, Eastern Cape; QLD, Queensland; NSW, New South Wales. Diversity metrics: *n*, number of samples per site; *A*, mean number of alleles per locus; *A_r_*, allelic richness; *A_pr_*, private allelic richness; *H_o_*, observed heterozygosity; *H_e_* unbiased expected heterozygosity; *F_IS_*, inbreeding coefficient; *F_IS-c_*, inbreeding coefficient corrected for null alleles; *D_est_*, Jost's estimator of actual differentiation; *F_ST_*, Wright's fixation index; *F'_ST_*, Wright's fixation index scaled to maximum possible value.
(PDF)

Table S3 **Polymorphic microsatellite primer pairs used in analysis, including fluorescent dye.** Microsatellite loci originally developed by Le Roux (nuclear) [42] and Weising and Gardner (chloroplast) [43]. Annealing temperatures used in PCR (T_a_), number of alleles (A), amplicon length in base pairs not including primers (L) and error rate per allele (E_a_) and locus (E_l_).
(PDF)

Table S4 **Site details, diversity and haplotype frequencies of all individuals used for chloroplast microsatellite analysis at three loci.** Population locations: KZN, KwaZulu-Natal; EC, Eastern Cape. Australian populations comprised of herbarium accessions falling into the geographic ranges of the mid-eastern population (P2) and south-eastern population (P1), based on clustering of the nuclear data as defined by structure [44]. Diversity measures: *N_i_*, number of individuals; *N_h_*, number of haplotypes (parentheses denote private haplotypes); *H*, Simpson's diversity index; *R_h_*, haplotypic richness (rarefied to *n* = 11).
(PDF)

Table S5 **Hypotheses tested in simulations.** These simulations were run in Resampling Stats Add-In for Excel v4.0 (statistics.com) with 10,000 repeat samples.
(PDF)

Table S6 **Major ports in eastern Australia used in simulations.** Name, location and size of ports in eastern Australia obtained from worldportsource.com.
(PDF)

Table S7 **Results of Kruskal-Wallis tests and Dunn's post-hoc tests.** Diversity metrics obtained from sites in South Africa (SA), South-Eastern Australian population (P1) and Mid-Eastern Australian population (P2). Diversity measures are *A*, mean number of alleles per locus; *A_r_*, allelic richness; *A_pr_*, private allelic richness; *H_o_*, observed heterozygosity; *H_e_* unbiased expected heterozygosity. *H_c_* is the test statistic *H* corrected for ties. Significance levels are indicated as *P*<0.05 = *; *P*<0.01 = **; *P*<0.001 = ***; *P*<0.0001 = ****.
(PDF)

Appendix S1 **Microsatellite loci evaluation methods and results.**
(PDF)

Appendix S2 **Microsatellite loci allele call data for all samples in genalex format.**
(XLSX)
