# Supplementary figures and images for: Genetic Bottlenecks in Time and Space: Reconstructing Invasions from Contemporary and Historical Collections
Source: PLoS One. 2014 Sep 5;9(9):e106874. doi: 10.1371/journal.pone.0106874 (PMC4156389; doi:10.1371/journal.pone.0106874)

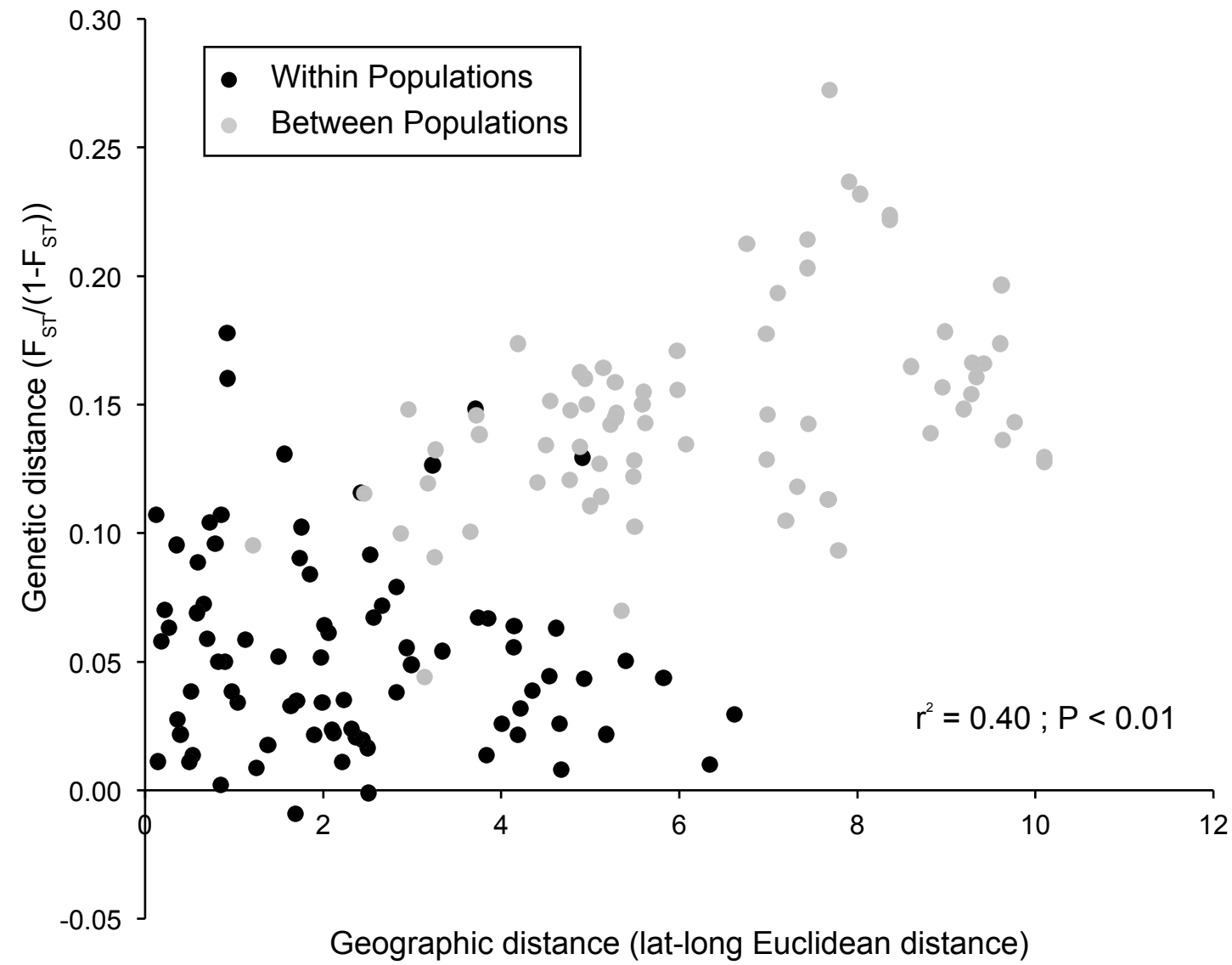

Supplement: Figure S1 — Isolation by distance between and within populations. Linearised FST values (FST/(1-FST)) regressed on lat-long Euclidean distance between pairs of sampled sites. Shading shows the effects of between and within population comparisons. Significant isolation-by-distance (Mantel's test P<0.01; r = 0.63) was detected across Australia as a whole. (PDF) [file pone.0106874.s001.pdf]
