# Supplementary material for: Genetic Bottlenecks in Time and Space: Reconstructing Invasions from Contemporary and Historical Collections
Source: PLoS One. 2014 Sep 5;9(9):e106874. doi: 10.1371/journal.pone.0106874 (PMC4156389; doi:10.1371/journal.pone.0106874)
Supplement: Table S1 — Senecio madagascariensis herbarium voucher details. (PDF) [file pone.0106874.s002.pdf]

Herbarium	Site	Latitude	Longitude	Voucher number	Ploidy
South African National Herbarium	Vryheid, KZN	S 27° 47' 4"	E 30° 48' 25"	JGR 1101	diploid
	Hluhluwe, KZN	S 27° 48' 48"	E 32° 24' 5"	JGR 1103	polyploid
	Denny Dalton, KZN	S 28° 17' 3"	E 31° 12' 42"	JGR 1102	diploid
	Ongoye, KZN	S 28° 54' 4"	E 31° 48' 38"	JGR 1106	diploid
	Tinley Manor, KZN	S 29° 27' 2"	E 31° 16' 58"	JGR 1107	diploid
	Pietermaritzburg, KZN	S 29° 38' 10"	E 30° 24' 8"	JGR 1117	diploid
	Summerveld, KZN	S 29° 48' 6"	E 30° 42' 35"	JGR 1115	diploid
	Durban, KZN	S 29° 51' 51"	E 30° 58' 56"	JGR 1108	diploid
	Richmond, KZN	S 29° 52' 58"	E 30° 16' 48"	JGR 1116	diploid
	Pennington, KZN	S 30° 22' 42"	E 30° 40' 34"	JGR 1109	diploid
	Port Edward, KZN	S 31° 2' 5"	E 30° 10' 44"	JGR 1114	diploid
Stellenbosch University Herbarium	Boesmansriviermond, EC	S 33° 41' 4"	E 26° 39' 12"	SA/SMAD/A2/01	diploid
Queensland Herbarium	Halfway Creek, NSW	S 29° 56' 6"	E 153° 5' 44"	AQ741268	diploid
