# Supplementary material for: Genetic Bottlenecks in Time and Space: Reconstructing Invasions from Contemporary and Historical Collections
Source: PLoS One. 2014 Sep 5;9(9):e106874. doi: 10.1371/journal.pone.0106874 (PMC4156389; doi:10.1371/journal.pone.0106874)
Supplement: Table S2 — Location details and diversity metrics of all sites sampled for nuclear microsatellite analysis. Population locations: KZN, KwaZulu-Natal; EC, Eastern Cape; QLD, Queensland; NSW, New South Wales. Diversity metrics: n, number of samples per site; A, mean number of alleles per locus; Ar, allelic richness; Apr, private allelic richness; Ho, observed heterozygosity; He unbiased expected heterozygosity; FIS, inbreeding coefficient; FIS-c, inbreeding coefficient corrected for null alleles; Dest, Jost's estimator of actual differentiation; FST, Wright's fixation index; F'ST, Wright's fixation index scaled to maximum possible value. (PDF) [file pone.0106874.s003.pdf]

**Population**	**Latitude**	**Longitude**	n	A	A_r	A_{pr}	H_o	H_e	F_{IS}	$F_{IS=c}$	D_{est}	F_{ST} **(±SE)***	F'_{ST} **(±SE)***
**South Africa (native)**													
Vryheid, KZN	S 27° 47' 4"	E 30° 48' 25"	20	10.9	6.5	0.065	0.599	0.839	0.264	0.055			
Denny Dalton, KZN	S 28° 17' 3"	E 31° 12' 42"	20	10.9	6.3	0.234	0.606	0.826	0.263	0.072			
Ongoye, KZN	S 28° 54' 4"	E 31° 48' 38"	20	11.0	7.2	0.124	0.629	0.871	0.269	0.059			
Tinley Manor, KZN	S 29° 27' 2"	E 31° 16' 58"	20	12.1	7.6	0.052	0.513	0.860	0.380	0.149			
Pietermaritzburg, KZN	S 29° 38' 10"	E 30° 24' 8"	20	12.6	7.2	0.194	0.678	0.845	0.185	0.095			
Summerveld, KZN	S 29° 48' 6"	E 30° 42' 35"	20	13.7	7.3	0.671	0.628	0.870	0.261	0.068			
Durban, KZN	S 29° 51' 51"	E 30° 58' 56"	20	12.9	6.8	0.029	0.614	0.855	0.258	0.177			
Richmond, KZN	S 29° 52' 58"	E 30° 16' 48"	19	12.3	6.7	0.187	0.693	0.860	0.196	0.026			
Pennington, KZN	S 30° 22' 42"	E 30° 40' 34"	20	11.1	6.1	0.304	0.624	0.819	0.263	0.034			
Port Edward, KZN	S 31° 2' 5"	E 30° 10' 44"	19	12.0	7.9	0.203	0.585	0.865	0.305	0.031			
Boesmansriviermond, EC	S 33° 41' 4"	E 26° 39' 12"	20	8.4	6.2	0.530	0.493	0.711	0.237	0.085			
Mean (±SE) South Africa			19.8	11.6	6.9	0.236	0.606	0.838	0.262	0.077			
			(±0.1)	(±0.6)	(±0.2)	(±0.061)	(±0.028)	(±0.015)	(±0.015)	(±0.014)			
Total South Africa			218	32.3	7.8	4.008	0.611	0.883	0.883	0.048	0.283	0.044	0.289
												(±0.013)	(±0.085)
**Australia (invasive)**													
**Far North Queensland**													
Malanda, QLD†	S 17° 30' 30"	E 145° 31' 08"	16	6.1	4.9	<0.001	0.561	0.722	0.722	0.033			
Herberton, QLD‡	S 17° 25' 44"	E 145° 26' 15"	20	5.9	4.4	<0.001	0.382	0.687	0.687	0.050			
**Mid-Eastern Australian population (P2)**													
Mount Glorious, QLD	S 27° 22' 37"	E 152° 50' 31"	20	5.9	4.0	<0.001	0.552	0.670	0.670	0.024			
Mitchelton, QLD	S 27° 24' 44"	E 152° 57' 45"	19	5.9	4.3	0.001	0.458	0.681	0.681	0.053			
Oxenford, QLD	S 27° 53' 23"	E 153° 18' 43"	20	6.1	4.6	0.007	0.431	0.677	0.677	0.056			
Canungra, QLD	S 27° 58' 0"	E 153° 8' 36"	20	7.4	4.6	0.036	0.446	0.698	0.698	0.037			
Lamington, QLD	S 28° 11' 7"	E 153° 6' 56"	20	5.9	4.2	0.002	0.508	0.686	0.686	0.055			
Halfway Creek, NSW	S 29° 56' 6"	E 153° 5' 44"	20	7.9	5.0	<0.001	0.524	0.775	0.775	0.095			
Mean ±SE P2			19.8	6.5	4.5	0.008	0.486	0.698	0.698	0.053			
			(±0.2)	(±0.4)	(±0.1)	(±0.006)	(±0.044)	(±0.022)	(±0.022)	(±0.009)			
Total P2			119	13.4	5.2	0.959	0.492	0.758	0.758	0.039	0.158	0.081	0.276
												(±0.019)	(±0.065)
**South-Eastern Australian population (P1)**													
Crescent Head, NSW	S 31° 7' 45"	E 152° 52' 50"	20	9.9	6.2	0.042	0.584	0.812	0.812	0.043			
Elizabeth Beach, NSW	S 32° 19' 49"	E 152° 32' 9"	20	10.3	6.5	0.129	0.611	0.775	0.775	0.037			
Hunter Valley, NSW	S 32° 36' 55"	E 152° 4' 45"	20	10.1	5.9	0.001	0.645	0.812	0.812	0.038			
Black Hill, NSW	S 32° 50' 20"	E 151° 37' 48"	20	10.6	6.3	0.096	0.621	0.816	0.816	0.040			
Raymond Terrace, NSW	S 32° 45' 58"	E 151° 44' 55"	20	10.9	6.8	0.002	0.575	0.726	0.726	0.095			
Jilliby, NSW	S 33° 11' 58"	E 151° 27' 58"	20	7.1	6.6	<0.001	0.489	0.683	0.683	0.046			
Illawarra, NSW	S 34° 31' 5"	E 150° 45' 1"	20	8.4	5.6	0.160	0.552	0.740	0.740	0.055			
Gerroa, NSW	S 34° 46' 45"	E 150° 49' 19"	19	10.0	6.6	0.048	0.565	0.855	0.855	0.082			
Tabourie Lake, NSW	S 35° 23' 37"	E 150° 26' 18"	20	7.3	5.2	<0.001	0.543	0.604	0.604	0.022			
Cooma, NSW	S 32° 19' 49"	E 152° 32' 9"	19	7.3	6.6	0.005	0.577	0.780	0.780	0.056			
Tilba, NSW	S 36° 14' 59"	E 150° 6' 14"	20	8.0	5.5	0.068	0.606	0.750	0.750	0.040			
Eden, NSW	S 37° 2' 29"	E 149° 54' 30"	20	9.4	6.6	0.064	0.671	0.814	0.814	0.027			

Mean ±SE P1	19.8<br>(±0.1)	9.1<br>(±0.5)	6.2<br>(±0.2)	0.051<br>(±0.016)	0.587<br>(±0.028)	0.764<br>(±0.022)	0.210<br>(±0.031)	0.048<br>(±0.006)			
Total P1	238	22.4	6.7	2.331	0.612	0.833	0.275	0.027	0.237§	0.049<br>(±0.010)	0.240<br>(±0.049)
Mean ±SE Australia	19.7<br>(±0.2)	8.0<br>(±1.3)	5.5<br>(±0.2)	0.038<br>(±0.011)	0.545<br>(±0.023)	0.738<br>(±0.015)	0.250<br>(±0.028)	0.049<br>(±0.005)			
Total Australia	393	23.5	6.8	2.997	0.568	0.843	0.335	0.038	0.420§	0.100<br>(±0.012)	0.422<br>(±0.051)
Mean ±SE South Africa & Australia	19.8<br>(±0.1)	9.3<br>(±0.3)	6.0<br>(±0.2)	0.105<br>(±0.028)	0.567<br>(±0.018)	0.774<br>(±0.012)	0.254<br>(±0.021)	0.059<br>(±0.006)			
Total South Africa & Australia	611	35.4	7.6		0.585	0.880	0.342	0.047	0.487§	0.100<br>(±0.013)	0.459<br>(±0.060)

*Jackknifed over all loci
†Site clusters with southern Australian population
‡Site clusters with mid Australian population
§Two Australian populations (Jilliby and Raymond Terrace) excluded from calculation due to missing data
