# Supplementary material for: Genetic Bottlenecks in Time and Space: Reconstructing Invasions from Contemporary and Historical Collections
Source: PLoS One. 2014 Sep 5;9(9):e106874. doi: 10.1371/journal.pone.0106874 (PMC4156389; doi:10.1371/journal.pone.0106874)
Supplement: Table S3 — Polymorphic microsatellite primer pairs used in analysis, including fluorescent dye. Microsatellite loci originally developed by Le Roux (nuclear) [42] and Weising and Gardner (chloroplast) [43]. Annealing temperatures used in PCR (Ta), number of alleles (A), amplicon length in base pairs not including primers (L) and error rate per allele (Ea) and locus (El). (PDF) [file pone.0106874.s004.pdf]

Marker	Locus	Forward primer	Reverse primer	T_a (°C)	*A*	*L* (bp)	E_a	E_l
**nSSR**	Se-116	CCTTCTGGTTGATTTGGCTAAGC(FAM)	AGAACTGCACATTTGAAGCCTG	48	22	370-432	0.000	0.000
	Se-136	CAAAGGTAGGATGATGTGAAGCTC(FAM)	TCTTGTTGGGTCAATGCTCG	51	74	257-358	0.069	0.083
	Se-138	ACTTCGTGGGCCATTCCAG(VIC)	CTTCCTGCATAACATCCACCAC	58	30	123-185	0.044	0.086
	Se-176(a)	AGCATAGTGCAAGCATGTTCG(FAM)	CTTTGATGTTGGCTGCAATGC	60	29	185-245	0.007	0.014
	Se-194	GTCGCAGTCACCGTCACTG(VIC)	GAGCAGCAGACAACGACAC	58	9	307-318	0.031	0.061
	Se-208	TTTTGGGCAGGCCATATCC(NED)	AGTGTCTCCACGGTTGTCG	55	61	264-360	0.020	0.040
	Se-220	AACTCGACCAGTCCTCAGC(NED)	GTCACACTGGAACCCAACTG	58	23	115-175	0.033	0.039
**cpSSR**	Ccmp1	CAGGTAAACTTCTCAACGGA(VIC)	CCGAAGTCAAAAGAGCGATT	50	3	159-161	0.053*	
	Ccmp2	GATCCCGGACGTAATCCTG(PET)	TCGTACCGAGGGTTCGAAT	50	3	217-219	0.043*	
	Ccmp5	TGTTCCAATATCTTCTTGTCATTT(FAM)	AGGTTCCATCGGAACAATTAT	50	2	112-115	0.000*	

*Error rate per locus and per allele are the same in haploid genomes
