# Supplementary material for: Genetic Bottlenecks in Time and Space: Reconstructing Invasions from Contemporary and Historical Collections
Source: PLoS One. 2014 Sep 5;9(9):e106874. doi: 10.1371/journal.pone.0106874 (PMC4156389; doi:10.1371/journal.pone.0106874)
Supplement: Table S4 — Site details, diversity and haplotype frequencies of all individuals used for chloroplast microsatellite analysis at three loci. Population locations: KZN, KwaZulu-Natal; EC, Eastern Cape. Australian populations comprised of herbarium accessions falling into the geographic ranges of the mid-eastern population (P2) and south-eastern population (P1), based on clustering of the nuclear data as defined by structure [44]. Diversity measures: Ni, number of individuals; Nh, number of haplotypes (parentheses denote private haplotypes); H, Simpson's diversity index; Rh, haplotypic richness (rarefied to n = 11). (PDF) [file pone.0106874.s005.pdf]

**Population**	***N_i***	***N_h***	***H***	***R_h***	**Haplotypes**													
					**A**	**B**	**C**	**D**	**E**	**F**	**G**	**H**	**I**	**J**	**K**	**L**	**M**	**N**
**South Africa (native)**																		
Vryheid, KZN	18	5(0)	0.771	3.945	5	–	1	–	–	2	7	3	–	–	–	–	–	–
Denny Dalton, KZN	20	7(1)	0.832	4.621	2	–	7	–	–	1	3	2	1	4	–	–	–	–
Ongoye, KZN	19	4(0)	0.573	2.397	12	–	4	1	2	–	–	–	–	–	–	–	–	–
Tinley Manor, KZN	14	2(0)	0.440	1.000	10	–	4	–	–	–	–	–	–	–	–	–	–	–
Pietermaritzburg, KZN	11	4(0)	0.691	3.833	6	–	–	2	–	1	2	–	–	–	–	–	–	–
Summerveld, KZN	19	6(0)	0.661	3.667	11	1	2	–	2	2	1	–	–	–	–	–	–	–
Durban, KZN	19	3(0)	0.433	1.561	14	–	4	–	–	1	–	–	–	–	–	–	–	–
Richmond, KZN	18	4(0)	0.471	2.179	13	1	3	–	–	–	1	–	–	–	–	–	–	–
Pennington, KZN	20	4(1)	0.626	2.358	2	–	11	–	–	–	–	–	–	–	6	1	–	–
Port Edward, KZN	17	5(0)	0.621	2.425	13	1	1	–	–	1	–	–	–	–	1	–	–	–
Boesmansriviermond, EC	20	3(0)	0.195	1.100	18	–	1	–	–	–	1	–	–	–	–	–	–	–
Mean ±SE South Africa	17.7<br>(±0.9)	4.3<br>(±0.4)	2.644<br>(±0.366)	0.574<br>(±0.054)														
Total South Africa	195	12(8)	3.331	0.659	106	3	38	3	4	8	15	5	1	4	7	1	–	–
**Australia (invasive)**																		
Mid-Eastern Australian population (P2)	95	5(2*)	0.316	1.451	78	–	9	–	–	–	6	–	–	–	–	–	1	1
South-Eastern Australian population (P1)	128	4(1†, 0*)	0.522	1.998	84	–	24	–	–	–	16	4	–	–	–	–	–	–
Mean ±SE Australia	111.5<br>(±16.5)	3.5<br>(±0.5)	1.725<br>(±0.274)	0.419<br>(±0.103)														
Total Australia	223	4(0)	1.808	0.442	162	–	33	–	–	–	22	4	–	–	–	–	1	1
Total South Africa & Australia	418	14	2.561	0.552	268	3	71	3	4	8	37	9	1	4	7	1	1	1

* Australia and South Africa; † Australia only
