# Supplementary material for: Genetic Bottlenecks in Time and Space: Reconstructing Invasions from Contemporary and Historical Collections
Source: PLoS One. 2014 Sep 5;9(9):e106874. doi: 10.1371/journal.pone.0106874 (PMC4156389; doi:10.1371/journal.pone.0106874)
Supplement: Table S5 — Hypotheses tested in simulations. These simulations were run in Resampling Stats Add-In for Excel v4.0 (statistics.com) with 10,000 repeat samples. (PDF) [file pone.0106874.s006.pdf]

**Null hypothesis**	***P***
Haplotypic composition of Australia in 1957 is a random sample of the diversity present in the native range	0.633
Haplotypic composition of Australia in 1987 is a random sample of the diversity present in the native range	<0.01
Haplotypes G and H were not present in the first 32 herbarium records due to a sampling effect	0.019
Haplotypes G and H were both first found within 35 km of a major port by chance	0.012
Similarities in haplotype composition between P1 and P2 can be explained by random independent sampling from the native range	0.813
