# Supplementary material for: Genetic Bottlenecks in Time and Space: Reconstructing Invasions from Contemporary and Historical Collections
Source: PLoS One. 2014 Sep 5;9(9):e106874. doi: 10.1371/journal.pone.0106874 (PMC4156389; doi:10.1371/journal.pone.0106874)
Supplement: Table S6 — Major ports in eastern Australia used in simulations. Name, location and size of ports in eastern Australia obtained from worldportsource.com. (PDF) [file pone.0106874.s007.pdf]

Port Name	Size	Latitude	Longitude
Port Botany	Medium	S 33° 58' 23"	E 151° 12' 54"
Port of Brisbane	Large	S 27° 22' 57"	E 153° 10' 10"
Port of Gladstone	Medium	S 23° 49' 48"	E 151° 15' 10"
Port Kembla	Medium	S 34° 27' 45"	E 150° 53' 58"
Port of Melbourne	Very Large	S 37° 49' 26"	E 144° 54' 40"
Port of Newcastle	Medium	S 32° 54' 27"	E 151° 46' 14"
Port of Sydney	Large	S 33° 51' 44"	E 151° 11' 33"
