# Supplementary material for: Genetic Bottlenecks in Time and Space: Reconstructing Invasions from Contemporary and Historical Collections
Source: PLoS One. 2014 Sep 5;9(9):e106874. doi: 10.1371/journal.pone.0106874 (PMC4156389; doi:10.1371/journal.pone.0106874)
Supplement: Table S7 — Results of Kruskal-Wallis tests and Dunn's post-hoc tests. Diversity metrics obtained from sites in South Africa (SA), South-Eastern Australian population (P1) and Mid-Eastern Australian population (P2). Diversity measures are A, mean number of alleles per locus; Ar, allelic richness; Apr, private allelic richness; Ho, observed heterozygosity; He unbiased expected heterozygosity. Hc is the test statistic H corrected for ties. Significance levels are indicated as P<0.05 = *; P<0.01 = **; P<0.001 = ***; P<0.0001 = ****. (PDF) [file pone.0106874.s008.pdf]

**Genetic Diversity Measure**	**Kruskal-Wallis test H_c**	**Dunn's post-hoc test result**
A	20.313****	SA > P1** & P2****
A_r	17.158***	P2 < P1** & SA****
A_{pr}	14.939***	SA > P1** & P2***
H_o	11.233**	P2 < P1* & SA***
H_e	16.741***	SA > P1** & P2****
