# Supplementary material for: Genetic Bottlenecks in Time and Space: Reconstructing Invasions from Contemporary and Historical Collections
Source: PLoS One. 2014 Sep 5;9(9):e106874. doi: 10.1371/journal.pone.0106874 (PMC4156389; doi:10.1371/journal.pone.0106874)
Supplement: Appendix S1 — Microsatellite loci evaluation methods and results. (PDF) [file pone.0106874.s009.pdf]

Appendix S1 – Microsatellite loci evaluation

Methods

Prior to scoring, all microsatellites were evaluated for consistency in banding pattern across individuals; any inconsistent loci were not scored and excluded from all subsequent analyses. For the nuclear microsatellite dataset, Linkage disequilibrium (LD) and deviations from Hardy-Weinberg (HW) equilibrium were investigated using GENEPOP on the web [1,2] and significance levels adjusted using sequential Bonferroni corrections [3]. The program MICRO-CHECKER [4] was used to examine large allele dropout, stuttering and null alleles as potential sources of error. Null allele frequencies were estimated using FREENA [5]. Genetic clusters in HW were determined using the program STRUCTURE v2.3.3 [6] using the admixture model. As the presence of null alleles introduces potential ambiguity around the true underlying genotype, we ran the program under two conditions; RECESSIVEALLELES set to 0 in which no ambiguity is assumed; and RECESSIVEALLELES set to1 where missing data is assigned as recessive to better account for null alleles [7]. To examine the effect of null alleles on F_{ST} estimates, a Mantel test with 9,999 permutations was carried out in GENALEX v6.4 [8] between the INA corrected and uncorrected F_{ST} estimates obtained from FREENA [5]. We used POWSIM v4.0 [9] to assess the power of our microsatellite dataset to detect genetic heterogeneity in Australia. Error rates were determined according to DeWoody et al [10]; specifically error rates per allele, per locus and over all loci were calculated for both the nuclear and chloroplast microsatellite datasets.

Results

Two nuclear loci were excluded due to inconsistent banding patterns, the remaining seven were polymorphic and used in further analyses. There was no evidence of large allele dropout in any loci. Stuttering was identified as a potential issue at one native and one invasive site for one locus. Significant LD was found for three different loci combinations, each at one of three different sites. All loci showing potential stuttering or LD were retained in the dataset as genuine stuttering and LD are expected to affect all sites equally. Significant deviations from HW equilibrium were found in 92 of 212 tests (43%), indicating heterozygote deficiency. Potential presence of null alleles was found in 80 of 182 tests (44%). Significant HW deviation corresponded with presence of null alleles in 90% of cases. The null allele frequency distribution was estimated to have a mean of 0.19, with 25% and 75% quartiles of 0.07 and 0.37 respectively. In STRUCTURE [6] there were no appreciable differences in optimum values of *K* or assignment of individuals to each cluster when RECESSIVEALLELES set to 0 or 1, therefore we only report results as per RECESSIVEALLELES set to 0. No loci were dropped due to presence of null alleles to minimise power loss, as when dealing with a low number of loci, it is generally preferable to account for null alleles rather than exclude loci [11]. Pairwise F_{ST} values corrected and uncorrected for null alleles were strongly and significantly correlated (r = 0.97, $P < 0.001$) suggesting that the effect of null alleles was similar across populations. Uncorrected F_{ST} values only are reported throughout the paper. Power analysis determined that our nuclear microsatellite dataset could accurately detect F_{ST} values > 0.01 ($P = 1$) indicating that our markers are adequate to detect the population structure in *S. madagascariensis*. Overall, the mean genotyping error

rates per locus and per allele were 0.047 and 0.029 respectively. Errors per locus and per allele were less in Australia (0.023 and 0.035 respectively) than in South Africa (0.035 and 0.060 respectively). Error rates for individual loci are listed in Table S3, Supporting Information.

References

1. Rousset F (2008) GENEPOP'007: a complete re-implementation of the GENEPOP software for Windows and Linux. Molecular Ecology Resources 8: 103-106.
2. Raymond M, Rousset F (1995) GENEPOP (Version 1.2): Population genetics software for exact tests and ecumenicism. Journal of Heredity 86: 248-249.
3. Hochberg Y (1988) A sharper Bonferroni procedure for multiple tests of significance. Biometrika 75: 800-802.
4. Van Oosterhout C, Hutchinson WF, Wills DPM, Shipley P (2004) MICRO-CHECKER: software for identifying and correcting genotyping errors in microsatellite data. Molecular Ecology Notes 4: 535-538.
5. Chapuis MP, Estoup A (2007) Microsatellite null alleles and estimation of population differentiation. Molecular Biology and Evolution 24: 621-631.
6. Pritchard JK, Stephens M, Donnelly P (2000) Inference of population structure using multilocus genotype data. Genetics 155: 945-959.
7. Falush D, Stephens M, Pritchard JK (2007) Inference of population structure using multilocus genotype data: dominant markers and null alleles. Molecular Ecology Notes 7: 574-578.

8. Peakall R, Smouse PE (2006) GENALEX 6: genetic analysis in Excel. Population genetic software for teaching and research. Molecular Ecology Notes 6: 288-295.

9. Ryman N, Palm S (2006) POWSIM: a computer program for assessing statistical power when testing for genetic differentiation. Molecular Ecology Notes 6: 600-602.

10. DeWoody J, Nason JD, Hipkins VD (2006) Mitigating scoring errors in microsatellite data from wild populations. Molecular Ecology Notes 6: 951-957.

11. Wagner AP, Creel S, Kalinowski ST (2006) Estimating relatedness and relationships using microsatellite loci with null alleles. Heredity 97: 336-345.
